# GenAPI: a tool for gene absence-presence identification in fragmented bacterial genome sequences

**DOI:** 10.1186/s12859-020-03657-5

**Published:** 2020-07-20

**Authors:** Migle Gabrielaite, Rasmus L. Marvig

**Affiliations:** Centre for Genomic Medicine, Rigshospitalet, University of Copenhagen, Copenhagen, Denmark

**Keywords:** Sequence analysis, Bacterial genomics, Evolution, Pangenomics, Gene presence-absence

## Abstract

**Background:**

Bacterial gene loss and acquisition is a well-known phenomenon which contributes to bacterial adaptation through changes in important phenotypes such as virulence, antibiotic resistance and metabolic capability. While advances in DNA sequencing have accelerated our ability to generate short genome sequence reads to disentangle phenotypic changes caused by gene loss and acquisition, the short-read genome sequencing often results in fragmented genome assemblies as a basis for identification of gene loss and acquisition events. However, sensitive and precise determination of gene content change for fragmented genome assemblies remains challenging as analysis needs to account for cases when only a fragment of the gene is assembled or when the gene assembly is split in more than one contig.

**Results:**

We developed GenAPI, a command-line tool that is designed to compare the gene content of bacterial genomes for which only fragmented genome assemblies are available. GenAPI, unlike other available tools of similar purpose, accounts for imperfections in sequencing and assembly, and aims to compensate for them. We tested the performance of GenAPI on three different datasets to show that GenAPI has a high sensitivity while it maintains precision when dealing with partly assembled genes in both simulated and real datasets. Furthermore, we benchmarked the performance of GenAPI with six popular tools for gene presence-absence identification.

**Conclusions:**

Our developed bioinformatics tool, called GenAPI, has the same precision and recall rates when analyzing complete genome sequences as the other tools of the same purpose; however, GenAPI’s performance is markedly better on fragmented genome assemblies.

## Background

Finding differences in the gene repertoire between bacterial clones is important to understand the genetic basis of differences in phenotypes such as virulence, antibiotic resistance, and metabolic capability. Also, phylogenetic analysis including information about the absence or presence of genes helps to inform about the pace and mechanisms by which genes are lost and acquired in bacterial populations [1]. Thus, genome-wide analysis of the gene presence or absence is necessary for a better understanding of bacterial evolution and adaptation.

There exist multiple open-source bioinformatics tools available for gene presence-absence identification. Each tool has its own set of advantages and limitations. Here we focus on tools that use assembled genomes as input. Some tools, e.g. PanSeq [2], are based on alignment of the query genome sequence to a reference genome to specifically test for the presence-absence of genes that are present in the given reference. Accordingly, this approach is limited knowing that prophage and plasmid genes constitute a major part of the variable bacterial genetic content [3]. Other tools, such as Roary [4], SaturnV [5], PanDelos [6], panX [7], BPGA [8] and EDGAR [9] construct a pan-genome from inputted genome assemblies and then determine the gene set that is present in each of the genome assemblies. The performance of this approach depends on the completeness of the queried genome sequences, and all previously mentioned tools are designed for analysis of near-complete genomes with only minor parts of genes missing due to sequencing and assembly imperfections. As a result, there is a need for analytical tools that are designed to account for highly fragmented assemblies (often the product of de novo genome assemblies based on short-read sequence data where genome assemblies have tens to hundreds of contigs) that else would result in a large number of false calls for gene being absent in the assembly.

**Fig. 1:**
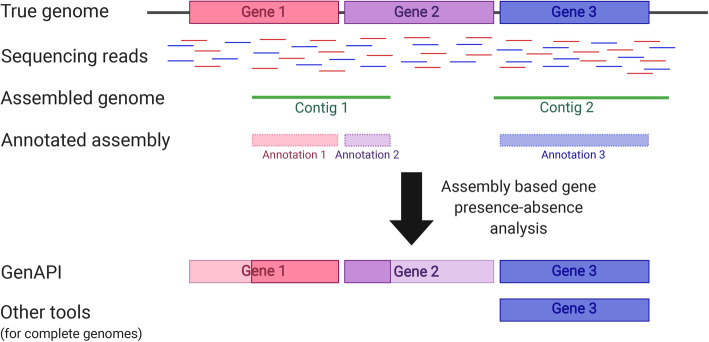
Illustration of how imperfect de novo assembly of genomes may lead to false gene absence calls. GenAPI is designed to compensate for partial gene assembly while other tools requires complete gene assembly

Here, we introduce GenAPI, a command line tool for identification and comparison of the gene content in bacterial clones of the same species. We specifically developed GenAPI to work successfully on annotated fragmented genome assemblies to account for cases when only a fragment of the gene is assembled or when the gene assembly is split in more than one contig (Fig. 1). The compensation for imperfections of sequencing and assembly proves to minimize the false gene absence calls while maintaining the true gene absence call rates.

## Implementation

**Fig. 2:**
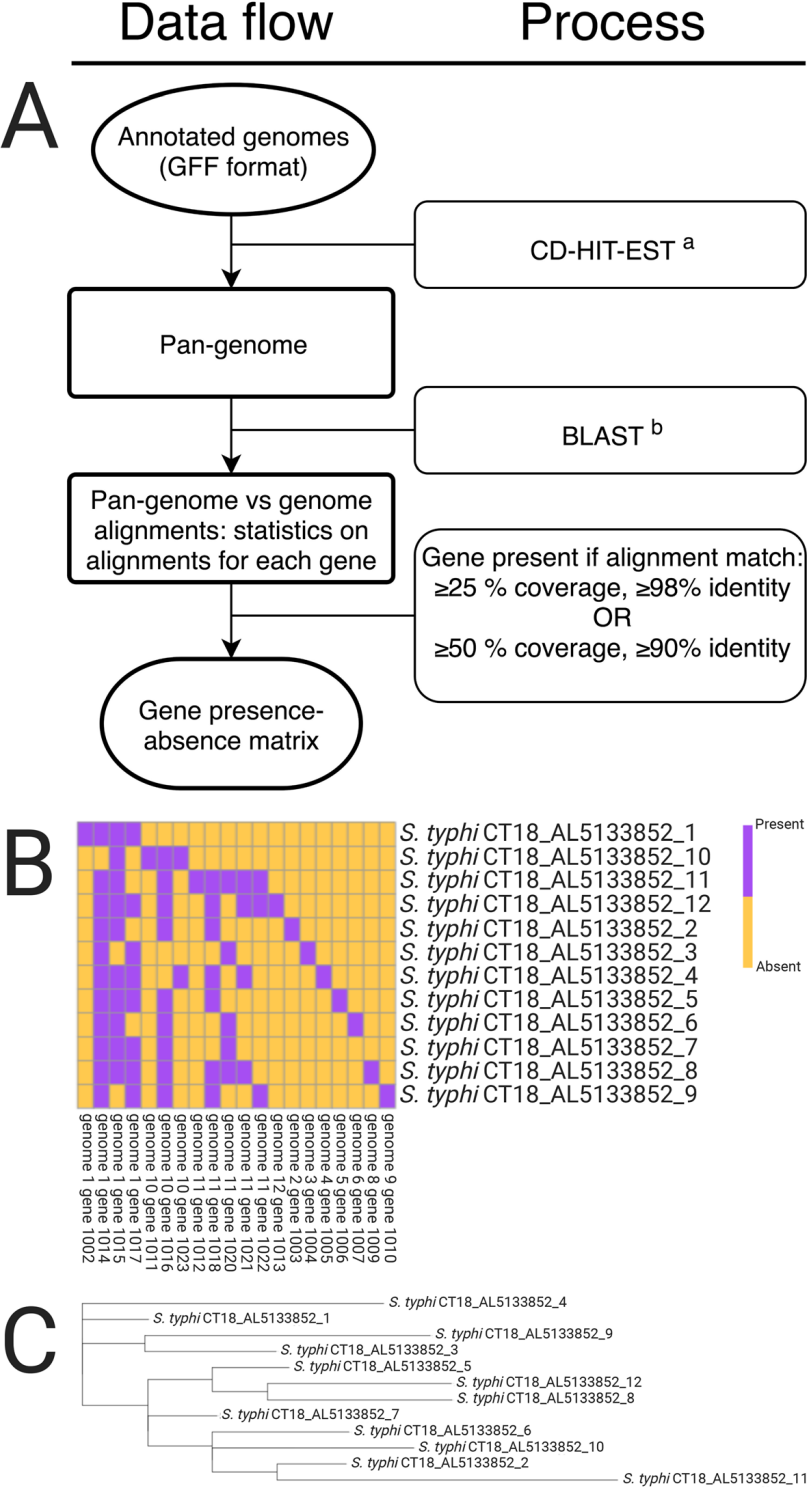
GenAPI workflow. **a** GenAPI data flow including the tools CD-HIT-EST [16]^a^, BLAST [10]^b^ or algorithms used in each step. GenAPI outputs (**b**) gene presence-absence matrix and (**c**) and phylogenetic tree based on gene presence-absence

GenAPI is designed to run in a Unix environment and requires BLAST+, CD-HIT and Bedtools software [10–12]. R with pheatmap library [13, 14] and RAxML [15] software are needed for optional gene presence-absence matrix visualization and phylogenetic tree generation. The step-wise workflow of GenAPI is shown in Fig. 2a where annotated genome sequence files are taken as input and the pan-genome (the total gene repertoire of the bacterial clones [17]) is generated by clustering the genes with CD-HIT-EST with default 90% identity and 80% sequence length overlap requirements. The 80% sequence overlap requirement allows incompletely sequenced or assembled genes to be clustered correctly. The longest gene sequence of each cluster becomes the gene representative of the respective cluster, and as such the pan-genome consist of a set of representative gene sequences. Gene clustering similarity thresholds may need to be changed if GenAPI is used for purposes or data that is markedly different from the present test, e.g. if genetically more distant bacterial genomes are compared. The best alignments between genes of the pan-genome and the BLAST database of each genome sequence are identified by performing all-vs-all sequence comparison. Nucleotide alignments were chosen to be used over amino acid alignments as it enables more specific evaluation of sequence identity (i.e., one amino acid can be defined by multiple codons) and includes RNA genes. Finally, the genes are defined as present or absent according to the user-provided thresholds (default: gene is present if best alignments have minimum 25% coverage with 98% identity or 50% coverage with 90% identity; more detailed method explanation is provided in Additional File 1: Figure S1).

The gene is defined as present even when it is only partly aligned but with high identity, this allows to compensate for sequencing and assembly imperfections while not compromising precision. The requirement for only 25% alignment coverage with nearly perfect identity is based on low likelihood of having a random, high-identity alignment of an average length gene (Fig. 1). In *Pseudomonas aeruginosa* PAO1 genome none of non-paralogous, non-rRNA genes had nonself alignments fulfilling these requirements when only 25% of the gene sequence was used during alignment (Additional File 1: Table S1). Nonetheless, genes shorter than 150 base pairs (bp) are recommended to be excluded from the gene presence-absence analysis as alignment of short gene sequences may produce unspecific alignments that may pass threshold for defining a gene as present (e.g. a 100 bp gene would be defined as present with an unspecific 25 bp alignment with 98% identity). Short genes are excluded from the analysis by default; however, this setting can be changed or turned off if needed. Furthermore, GenAPI is developed for identification of gene sequences that are completely lost or acquired in the genome, therefore it will not identify copy number changes of multicopy (paralog) genes—including pseudogenes—or partial gene deletions. To address these issues, long-read whole genome sequencing is recommended [18].

GenAPI outputs several files: (1) a pan-genome file, (2) a gene presence-absence matrix with information on the presence of each gene in each of analyzed genome sequences, and (3) a list of best alignment statistics for each analyzed genome. Furthermore, a heatmap visualization of gene presence-absence across all genomes and a maximum-likelihood phylogenetic tree based on gene presence-absence information are implemented in GenAPI (Fig. 2b-c).

## Results

We evaluated the performance of GenAPI on three test datasets with genome assemblies; two simulated datasets with in silico introduced variation in gene content (*P. aeruginosa* (this study) and *Salmonella typhi* [4] datasets, respectively), and one real dataset with known gene deletions (*Escherichia coli* experiment [19]). Furthermore, we compared the performance of GenAPI against Roary, SaturnV, PanDelos, BPGA, panX and EDGAR which are tools developed for similar but not identical purposes. None of the tools which we compared against were developed for fragmented genome assemblies; however, fragmented genome assemblies are most often the output from genome sequencing experiments, and there are no available tools developed for gene presence-absence analysis in fragmented genome assemblies.

If no insertions/deletions are introduced to the genomes, all genes in all analyzed genomes will be present; therefore, only gene absence was measured to evaluate the performance of the tools. The results of the predicted gene absence (recall, precision and F1 scores) are shown in Table 1. F1 measure was calculated in order to summarize precision and recall scores as it is a harmonic average of these two measures. Truth for gene absence was known for all three datasets. Genes which were correctly predicted to be absent were defined as true positive (TP) gene absence; genes incorrectly predicted to be present were defined as false negative (FN) gene absence; and genes incorrectly predicted to be absent were defined as false positive (FP) gene absence. Recall, precision and F1 scores were calculated by using the following formulas:

$$ Recall=\frac{TP}{TP+ FP};\kern0.5em Precision=\frac{TP}{TP+ FN}\kern0.5em F1=2\bullet \frac{Precision\bullet Recall}{Precision+ Recall} $$

**Table 1:** Recall, precision and F1 score of GenAPI, panX, BPGA, Roary, PanDelos, EDGAR and SaturnV using simulated and real datasets

		GenAPI	panX	BPGA	Roary	PanDelos	EDGAR	SaturnV
*S. typhi*^a^	Precision	1	1	0.93	1	NA	1	1
	Recall	1	1	1	1	NA	1	1
	F1	1	1	0.97	1	NA	1	1
*P. aeruginosa*^b^	Precision	0.91	0.38	0.39	0.35	0.43	0.18	0.18
	Recall	1	1	0.94	1	1	1	1
	F1	0.95	0.55	0.55	0.52	0.60	0.31	0.31
*E. coli*^c^	Precision	0.95	0.47	0.26	0.23	0.24	0.12	0.09
	Recall	0.98	1	0.88	1	0.71	1	1
	F1	0.97	0.64	0.40	0.38	0.36	0.21	0.17

^a^ simulated *S. typhi* dataset with complete genome sequences used in Roary publication [4]; ^b^ simulated *P. aeruginosa* dataset with partly assembled gene instances; ^c^ real *E. coli* dataset [19]

For simulated *P. aeruginosa* and long-term *E. coli* experiment [19] datasets Prokka version 1.11 [20] was used for gene annotation. Sequencing reads from both datasets were assembled using SPAdes version 3.10.1 software with default parameters [21] (*P. aeruginosa* and *E. coli* genome assembly statistics are reported in Additional File 1: Table S2 and Table S3, respectively). Information about known deletions in *E. coli* experiment dataset was obtained from Barrick et al. (2009) [19] study (Additional File 1: Table S5). *S. typhi* dataset was preprocessed as described by Page et al. (2015) [4]. All tools were tested with default parameters, with an exception for Roary for which paralog splitting was disabled since the other tools do not split identical sequences (paralogs), and Roary performance is better on draft genomes without splitting the paralogs. Default parameters were chosen assuming that they are the most optimal parameters for the tool. Sequencing reads for *P. aeruginosa* simulated dataset were simulated using ART version 2.5.8 software [22] using default parameters and the following settings: (1) MiSeq v3 sequencer, (2) 150 bp paired end sequences, (3) 500 bp fragment sizes with standard deviation of 10 and (4) 100X average genome coverage.

First, all tools except PanDelos were tested on the same simulated dataset of *S. typhi* that was used for the evaluation of Roary in its own publication [4]. PanDelos was excluded from the analysis as the tool did not finish the analysis in 24 h since its start. The six included tools identified all 181 instances where genes were absent from genomes (GenAPI did not include one gene as it was shorter than the default 150 bp gene length requirement) (Additional File 1: Table S4). BPGA additionally made 12 false calls of gene being absent. The other tools did not falsely call any genes to be absent.

Second, we simulated sequencing reads for a dataset of 8 *P. aeruginosa* genome sequences with known deletions in order to test the tools on data representing fragmented genome assemblies [23]. In total, there were 49 deleted genes (Additional File 1: Table S6). All tools except BPGA, which missed 3 deletions, correctly identified all 49 absent genes. However, false positive deletion calls were made by all tools. 90, 226 and 80 genes were false positively called as absent by Roary, SaturnV and BPGA, respectively. PanDelos and panX performance was better and resulted in 66 and 72 genes to be false positively called as deleted, respectively. On the other hand, only 5 false positive deletions were predicted by GenAPI (Additional File 1: Table S4).

Finally, we tested the tools on a dataset of 6 genome sequences with 102 known deletions (Additional File 1: Table S6) from the *E. coli* long-term evolution experiment performed by Richard Lenski [19]. Roary, SaturnV, EDGAR and panX called all true deletions, and GenAPI closely followed these tools except missing two copy number changes of homologous prophage genes. BPGA and PanDelos failed to identify 12 and 30 true deletions, respectively. The number of false positive calls of gene deletions was observed to be high for Roary, SaturnV, panX, PanDelos, BPGA and EDGAR (115–1009 false positive deletion calls) in contrast to GenAPI (5 false positive calls) (Additional File 1: Table S4). F1 scores from Table 1 show a summary of how well each tool performs with each of the datasets.

## Discussion

While there exist tools for bacterial gene content identification and comparison, our developed tool—GenAPI—was specifically designed for the analysis of fragmented, closely related genome sequences. Fragmented genome sequences are often the product of de novo genome assembly based on short-read sequence data, and GenAPI will therefore be suitable for use in multiple studies including bacterial genome sequence data.

Here we tested the performance of GenAPI on three different datasets to show that GenAPI has a high sensitivity while it maintains precision when dealing with partly assembled genes in both simulated and real datasets. While all tested tools except BPGA and PanDelos are excellent at identifying true gene absence (i.e. high sensitivity), only GenAPI has a low rate of false positive gene absence calls in fragmented genomes (i.e. high precision). The higher precision of GenAPI could be explained by the lower requirement for sequence alignment length which is conditional to a high alignment identity. The other tools included in the study have high requirements for sequence alignment length which does not cause any false gene absence predictions in simulated complete genome dataset (*S. typhi* dataset); however, it markedly affects the precision when applied to fragmented simulated (*P. aeruginosa*) and real (*E. coli*) datasets (Table 1, Additional File 1: Table S4). Similar observation has been made for Roary by Sieber et al. [24].

## Conclusions

We have shown that none of the other popular gene presence-absence identification tools are well suited for analysis of fragmented genome assemblies and the performance of GenAPI is better when analyzing fragmented genomes.

### Availability and requirements

**Project name:** GenAPI.

**Project home page:**https://github.com/MigleSur/GenAPI

**Operating system(s):** UNIX

**Programming language:** Bash

**Other requirements:** BLAST 2.6.0+ or higher, CD-HIT 4.6.1 or higher, Bedtools 2.26 or higher

**License:** GPLv3

**Any restrictions to use by non-academics:** None

## Supplementary information

**Additional file 1: Table S1.** Best non-self hit of all PAO1 reference genome BLAST experiment where 25% of the gene was blasted against the whole PAO1 genome. Only genes longer than 150 bp are reported, only the alignments with 98% identity and minimum 25% gene length were reported. rRNA genes were excluded from report as their sequences are identical. Paralog sequences were defined with BLASTP. **Table S2.** Assembly statistics of the simulated *P. aeruginosa* dataset reads. **Table S3.** Assembly statistics of the *E. coli* dataset reads. **Table S4.** Performance test of GenAPI, Roary, PBGA, PanDelos, panX, EDGAR and SaturnV using (a) a simulated *S. typhi* dataset with complete genome sequences used in Roary publication [4], (b) a simulated *P. aeruginosa* dataset with partly assembled gene instances (fragmented genome sequences) and (c) a dataset from the long-term experiment with *E. coli* [19]. TP – true positive gene deletion calls (true positive absent genes); FP – false positive gene deletion calls (false positive absent genes); FN – false negative gene deletion calls (false negative absent genes). TP, FN, and TN were counted only for genes that showed variation between genome sequences to avoid inflation of numbers from large amount of non-variable gene sequences. (d) As one of the absent genes was shorter than 150 bp, it was excluded from GenAPI analysis; when the requirement for the minimum gene length was reduced to 100 bp, all 181 absent genes were successfully identified. **Table S5.** List of known absent genes in simulated *P. aeruginosa* dataset [23]. 0 - gene is absent, 1 - gene is present. **Table S6.** List of known deleted genes and their prokka annotations in *E. coli* dataset [23]. **Figure S1.** Detailed scheme of the GenAPI workflow.

## Data Availability

The datasets generated and analysed during the current study are available in the test_samples directory in GenAPI repository, https://github.com/MigleSur/GenAPI/; The datasets used and analyzed during the current study are available from the corresponding author on reasonable request.

## Abbreviations

DNA: Deoxyribonucleic acid
FN: False negative
FP: False positive
RNA: Ribonucleic acid
TP: True positive
